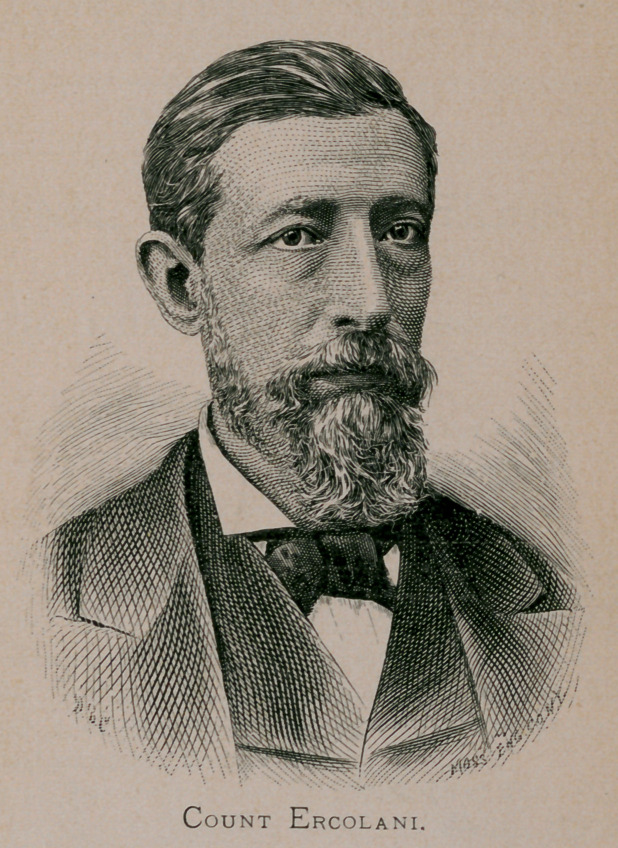# Eulogy on Count Ercolani*Delivered before the Anthropological Society of Washington, May 19th, 1885.

**Published:** 1885-10

**Authors:** Elmer R. Reynolds

**Affiliations:** Member of the Anthropological Society of Washington, Etc.


Count Ercolani.

### THE JOURNAL

OF

### COMPARATIVE MEDICINE AND SURGERY.

VOL. VI. OCTOBER, 1885. No. 4.

### ORIGINAL COMMUNICATIONS.

### ART. XXII.—EULOGY ON COUNT ERCOLANI.*

BY ELMER R. REYNOLDS, M.D.,
Member of the Anthropological Society of Washington, Etc.

"*Hector's love in Lethe cannot die.*"—HOMER.

Giovanni Battiste Ercolani, Count of the Holy Roman Empire, a Knight pre-eminently "*sans peur et sans reproche,*" was born in the renowned etruscan city of Felsina, December 23d, 1817. His father, Count Paul Leone Ercolani, was an eminent jurist of Bologna. His mother, the Countess Rosa Alba Celestina Lisi, was a lady distinguished for her social and intellectual accomplishments.

The origin of the noble house of Ercolani—or Hercules—can be traced to the war-like days of the old Roman Empire. Since then the name has been borne by chevaliers, barons, counts and princes. The church also has been represented by this family whose members have borne high sacerdotal rank. Finally, the name of Ercolani is inscribed upon the charters and in the annals of every province in Italy, and always with renown.

---

* Delivered before the Anthropological Society of Washington, May 19th, 1885.

Count Ercolani, of Bagna-Cavallo, the late distinguished scientist, entered the Royal University of Bologna at an early age. Here he soon graduated with uncommon honors. In 1836 he took his degree as Doctor of Medicine, and subsequently that of Doctor of Surgery and Veterinary Medicine. In 1837, while still in his twentieth year, he was appointed Assistant Professor of Anatomy and Veterinary Medicine, under the celebrated veterinarian, Antonio Alessandrini.

The first work of a practical nature that he undertook was the reorganization and enlargement of the Museum of Comparative Anatomy. This he lived to enrich by his systematic co-operation with kindred institutions in all quarters of the globe.

Although in those days a physician of the two schools of medicine, Count Ercolani declined to enter upon the practice of his profession, from which we may assume that these laborious studies had been undertaken for the purpose of aiding his investigations in the realm of natural history.

The first product of his biological research was published by the Bolognese Academy of Science, in 1842. This was his "Memoir on the Transmission of the Glanders from the Brute to the Human Family." This work, which had involved an immense amount of patient microscopic research, met with an immediate and cordial recognition, and led to the author's election as a member of many of the distinguished academies of Europe. During this period he also assisted in editing the "Medical Science Bulletin," and the "Annals of Natural Science," which were published under the supervision of Prof. Alessandrini.

Ercolani's numerous scientific investigations, and his original and profound deductions thereupon, were sufficient to rank him as a man of extraordinary genius, whose closer acquaintance and friendship were well worth seeking by the scientists of Europe. While his researches were chiefly of a biological nature, he nevertheless did not confine himself exclusively to this field, for his intimacy with such statesmen as Count Cavour and Marco Minghetti led him to embrace the study of Politico-Social Philosophy, a familiarity with which was of great importance to him during his subsequent Parliamentary career.

In 1843 he united himself in marriage with Miss Carlotta Sarti, of Bologna. Of this union two daughters were born, but one only reached the years of womanhood.

Previous to 1848, although conversant with the troubled and ever-shifting political affairs of the Peninsula, Ercolani had felt reluctant to identify himself with any issue that would withdraw his mind from his favorite pursuit; but, after Pius the IX. ascended the Papal throne, all religious scruples were cast aside, and thenceforth Ercolani boldly avowed his long dormant principles respecting the necessity for Italian unity, and more especially his utter hostility to foreign rule or interference. His active political career may be said to have commenced with the Austrian invasion of 1848. His service in any capacity whatever was now tendered to the general government, and in consequence his patriotism and special acquirements were recognized in his selection as Secretary of the Committee of Public Health which co-operated actively with the national party during that critical period.

Towards the end of 1848, he was transferred to Rome as Secretary to the Supreme Board of Public Health, whose duties he continued to discharge until 1849, when he was elected Member of the National Parliament. While serving as a member of the *Constituente Assembly* under the new Roman Republic, Ercolani directed his most earnest efforts towards the final unification of the several Italian provinces under the sway of one native king. That he was opposed to any form of government but a monarchy, and that his actions at this date were based upon the law of political expediency, is shown in a recent letter to me from Prince Camporeale, of Palermo, in which he says: "In the Roman Parliament (Constituente Assembly), Ercolani was strongly opposed to the establishment of the Republic, thinking that at this time it would have been pernicious (and so it was) to Italy. He showed great energy in opposing all the exaggerated proposals of the Mazzinian party. L. C. Farini, in his 'History of the Roman State,' praises him much. It is true that he took part in the defence of Rome from the first day the foreigners attacked the Republic. From this time Ercolani saw nothing but the necessity of saving the honor of the nation."

Immediate and harsh proscription followed upon the downfall of the new Latin Republic, and, as was to be expected, the name of Ercolani occupied a prominent place in the long list of those against whom vengence had been decreed. During this dangerous period, while monarchist and republican alike were seeking safety in the neutral states, Ercolani made his escape from Rome and sought refuge in the mountains of Bologna. Here he wandered about suffering the severest hardships from hunger and exposure in his efforts to elude the Papal *gendarmerie* who had been dispatched thither to affect his capture or death. In the winter of 1849 he succeeded in escaping from the mountains, and threw himself upon the protection of the Grand Duke of Tuscany. After residing for a time in Pistoia, he found it expedient to change his residence to Florence, where he was comforted by the presence of his family. It was while partaking of a brief repose in this city that he commenced his celebrated "Research Historic and Analytical on the Writers of the Veterinary Art."

His residence in Florence was rudely terminated by the action of the Papal Court, for, in the language of his eulogist, "It seemed intolerable that one who had combatted the majesty of the Pope, should find sanctuary in a neighboring Catholic state, and so strong was the remonstrance from Rome that the Grand Duke was compelled to expell the patriot from his territory on twenty-four hours notice."

Quitting Tuscany under the brief and unceremonious safeguard of the Grand Duke, Count Ercolani made his way northward into Piedmont, which was now the only place in the Peninsula where the Italian flag afforded protection. He arrived in Turin early in 1851, and here, in this palatial city, shadowed by the icy domes of the Alps, his fortune reached its most distressing and pathetic stage. As a mournful exile, resting under the all-powerful ban of the Church, with his lordly estates and all other earthly means sequestered, he was doomed to drink "the wormwood and the gall," and to learn in the most sorrowful manner

> "How hard it is to climb the stairs of others,
> And eat the salt of others on his bread."

This stringent condition of affairs may seem hard to understand, yet it should be borne in mind that many thousands of political refugees had claimed sanctuary in Turin, and among these, multitudes were without food or shelter, and, although the public and private benevolence of the city were taxed to their utmost limits, it was still a matter of very grave solicitude to relieve the most urgent cases as well as to furnish congenial employment for so vast and unforseen an increase in the civic population.

In these sombre hours the genius of Count Ercolani could not be ignored, for his eminent acquirements, and the adverse fortune which had driven him hither, gave him a two-fold claim upon the consideration of the patriotic Piedmontese, and were the means of obtaining for him the position of "substitute," or, Assistant Professor in the veterinary school of that city. Previous to this appointment, however, the citizenship of Piedmont had been conferred on him by a special decree of the king.

Count Ercolani now resumed his researches in natural history, which were directed chiefly towards the lower and more complex groups of animal life. These exhaustive and conscientious explorations into many of the slighted by-ways of nature, were the media of developing elaborate facts not hitherto recognized by the scientific world, and gave him a prestige in Italy similar to that won by Darwin among the Anglo-Saxon people. In 1852 he issued the first volume of his "Researches Historic and Analytical on the Writers of the Veterinary Art," the second and final volume of which was published in 1854. This was then, and is still, considered one of the most profound works of its scope ever issued in the south of Europe. During this period he also assisted in founding the first journal of veterinary medicine ever issued in Italy. In this work he was ably assisted by Prof. Carlo Lessoni, of Turin.

In 1855, during the cholera epidemic in Salluggio, Ercolani generously abandoned his professional duties and hastened into the infected district where he gave his whole attention to the relief of his fellow-countrymen until the disappearance of the disease. This, so far as I am informed, was the only instance in his career where he made a practical application of his knowledge of medicine.

In such profound respect was he now held by King Victor Emanuel that upon the reorganization of the educational establishments of Piedmont, Count Mamiani, the Minister of Public Instruction, was directed to promote him to the Directorship of the Veterinary School of Turin; thus advancing him from the lowest to the senior professorship. Contrary to the general rule, this flattering advancement met with the approbation, not only of the populace, but of the several faculties themselves.

In the winter of 1861 a sorrowful loss fell upon the now prosperous professor, and threatened for a time to deprive the world of his further career of usefulness. This event was the untimely death of his daughter Cæsarina, who suddenly yielded her life under an attack of parturition fever. The indescribable anguish and desolation which followed this bereavement induced Count Ercolani to resign his appointment, and, as political changes had at last removed the bar of expatriation, he resolved upon returning to his native city. In consequence, Turin lost her illustrious adopted son, and Ercolani once more set foot in Bologna after an exile of fourteen years.

Ercolani's resolution to retire from professional pursuits was of no avail, for upon arriving in his ancient home, the Minister of Public Instruction hastened to appoint him Professor of Pathological Anatomy in the Veterinary School of the Royal University. This appointment was followed by others which were both flattering and substantial. He was unanimously elected Rector of the Royal University, President of the Medico-Surgical Society, Permanent Secretary of the Academy of Sciences, Member of the Superior Council of Public Health, President of the Agrarian Society, Member of the Provincial Sanitary Commission of Bologna, and, for the second time, Member of the National Parliament, to which he was subsequently re-elected no less than three times. Among other important movements, he now proceeded to reorganize the School of Veterinary Medicine, which by the liberality of the Province and ample government grants, soon arose to be the first of its kind in Italy, and probably the most celebrated in the world. He also enlarged and greatly enriched the Anatomical Museum founded by Alessandrini, and, in addition he provided for a systematic and thorough course of Veterinary clinical lectures.

Ercolani may not have been the first to teach the doctrine, but he was the first to insist that a correct knowledge of Veterinary Medicine was as essential to the prosperity of the Kingdom, as that pertaining to human maladies. He strove to elevate and dignify this long neglected science, and finally succeeded in establishing a law which required candidates for veterinary honors to possess the same qualifications that were necessary to admit them into the regular universities in the Kingdom. Had he accomplished nothing else in life, this alone would have marked his grave for ages. In bearing witness to Ercolani's literary productions, it is enough to say that he was the author of no less than one hundred and thirty-six distinct memoirs embracing anthropology, biology medicine, and a vast acquaintance with other lofty subjects. Now, when it is considered that many of these productions fill large quarto volumes overflowing with original discoveries and deductions, we are permitted to form an opinion as to the unbounded resources and ceaseless industry of his mind. To specialize a few of his acquirements, it is stated that "He was a decided microscopist * * * and a passionate follower of both Medicine and Surgery, as well as of Natural History; as shown by his numerous discoveries in the fields of normal and pathological histology; of comparative tetratology; of elmintology; and of pathological anatomy and embryology." His numerous discoveries were not presented to the world until they had been subjected to the most penetrating experiments and comparisons known to modern science; then if his deductions were combatted, he sustained them with the same intellectual vigor that had characterized their patient and laborious production. An illustration of this nature is shown in his learned controversy with the celebrated Professor Kölliker of Wurzburg, Bavaria. On the contrary, however, if any of his theories were shown to be at variance or contrary to more recently discovered facts, or logical hypothesis, he yielded at once in the most graceful and liberal manner. An instance of this character is referred to by him in one of his letters to his distinguished American translator and correspondent, Dr. Henry O. Marcy, of Boston. In this communication he states that certain objections of Professors Albini, Pallidini, and Ohel, had been of decided advantage, inasmuch as they stimulated him to undertake supplementary investigations which developed valuable and previously unsuspected features in embryonic life.

Ercolani was equally as loyal to the rights of other authors from whom, in his personal judgement, the full credit of prior discovery had been withheld. This fact is shown in one of his memoirs wherein he "avenges" to Senator Carlo Ruini, of Bologna, the honor of having been the first to discover the circulation of the blood. "Noteworthy among his many works," says Prof. Cocconi, "are his patient '*Researches on the Genetic History of the Trematodic Worms, and the Adaptation of their Species to the Surrounding Fluid,*' in which is shown his great skill as an observer and naturalist. "'*The Formative Process of the Osseous Callus in the Different Fractures of the Bones of Men and Animals;*' '*The Interior Structure of the Tendinous Tissues, and that of Fibrous Tissues;*' '*The Transformation of the Histological Element in the Animal Organism;*' and other works on minute anatomy, attest his skill as a Histologist. But the work that obtained most attention from the scientists of both continents consisted of a series of memoirs regarding '*The Intimate Structure of the Placenta in Woman Compared with that of other Animals.*' In these observations he was led to determine the unity of the anatomical type, and the nutritive functions of the fœtus in all the vertebrates." It was the latter work which led to Ercolani's controversy with Prof. Kölliker, the results of which form an entire volume of new facts and masterly deductions which might have remained dormant had not the antagonism of the great German anatomist drawn them into existence.

As to the value of these discoveries, as well as to a brief statement respecting Ercolani's rank as a scientist, I take the liberty of quoting from Dr. Marcy, to whose discrimination the professional men of England and America are principally indebted for their knowledge of this author's ripest production. "The opinions, at a greater or less length, of this distinguished scientist, are quoted in nearly all the modern text-books on the subject, and yet so indifferently in many instances, and even erroneously, that it is evident that authors have not familiarized themselves with the elaborate and painstaking efforts of Professor Ercolani. * * * So simple are these demonstrations and the truths derived therefrom; so radically different are his teachings from the time-honored views still held and so generally taught, that I have felt the medical profession and students of natural history would gladly avail themselves of the opportunity of carefully examining these original investigations. Especially have they seemed to me valuable because of the attention which the study of the human placenta has received of late by many careful observers, and still more so since their conclusions are by no means unanimous. * * * By these researches is opened an almost unexplored field in the pathology of gestation, this, too, is essentially the medical and more practical side of the subject. In the abnormal development of the placenta, and in the modification of the nutrition of the fœtus, will be found causes hitherto unknown of embryonic disease and arrest of intra-uterine gestation. Therefore it will be seen that the work of the Bolognese Professor has an intimate connection and bearing upon anatomy and physiology, chemistry and pathology, embryology and anthropology, biology and obstetrics. It destroys ancient and classic errors; it demonstrates an important new anatomical fact; it teaches a new physiological function, and clearly shows a simple and fundamental plan of embryonic life. The evident impartiality of the author, as shown in his numerous observations, the multiplicity of facts produced, the modest and conscientious expression of opinion, * * * appeal to the unprejudiced reader, and carry conviction that the deductions presented are the results of thoughtful labor, and not preconceived theories which he has endeavored to demonstrate. The establishment of such facts will cause the name of Ercolani to be classed with the great benefactors of science and be handed down to coming generations, honored alike with Eustachius, Malpighi, Morgagni, and other distinguished anatomists of the early Italian School."

Reference has already been made to some of the high offices to which Count Ercolani was elected by his admiring countrymen. These however were not all the compliments which were bestowed upon him, for a man of such supreme abilities could not be overlooked by those in authority; hence we find that he was frequently called to Rome to confer with the Royal Government for the purpose of organizing or perfecting plans of national utility, which only men of his judgement and superior training could successfully undertake. "He was selected as a Member of the Royal Commission for the Publication of the Classics in the Province of Emilia; and all the Universities of the Kingdom, without distinction between medical and veterinary twice chose him as their representative in the Supreme Council of Public Instruction."

That King Victor Emanuel fully appreciated Ercolani's patriotism and his devotion to the literary advancement of the Kingdom is shown by the gracious advancement of the latter to the several distinguished orders of Knighthood. He was created Chevalier of the Royal Order of Civil Merit of Savoy, Commander of the Royal Maurizian Order, and Knight of the Crown of Italy. "Ercolani was worthy of all these honors," says Prof. Cocconi. "He did not seek them, nature having constituted him modest and disinterested. In manner he was affable towards all, and especially so with the young. He was not rigidly bound to routine, although he was scrupulous in the observation of his own professional duties. He was beloved by the youth whom he had as pupils for more than thirty years. He was cordial with friends and inferiors, and strove for the greatest advantage of all without slighting the interests of any."

Somewhere in one of Count Ercolani's works I remember having seen a passage to the effect that "The laborious conquests of human learning are never lost." This simple but pertinent expression has been verified abundantly by the mutations of three thousand years, and seem particularly applicable to this author's sublime life and works. It may transpire, however, that some of his works will not survive intact. New discoveries in the same boundless field may modify certain of his brilliant hypotheses, and, moreover, it may be shown that some particular features of his research lack elements now unsuspected, but, nevertheless, essential to their ultimate harmonious adjustment; or perhaps some of his masterly deductions may prove to be too free a rendition of what he undertook to interpret from the marvellous cryptology of nature. All these considerations will be "weighed in the balance," and Time will do him no injustice, but will touch these labors with a gentle hand, even as a harper stretches his open palm upon the quivering wires to modify their melodius cadence. Finally, it seems that Ercolani's works are both worthy and certain of earthly immortality, for so far as human wisdom goes, he builded in accordance with immutable laws.

A study of Ercolani's life, from the abundant memoirs, manuscript material and letters at my command, discloses that the most potent factors in his character were his abiding love and true-hearted sympathy for humanity. He was a nobleman, so born under the decree of a by-gone Pontiff; yet *noble* he was even without ancestral rank, for so he came fresh from the hand of God, and so he lived through a brilliant, but somewhat sorrowful life. All he claimed or aimed to be was simply a man, and with men he strove for his daily bread. Hence his works and noble brotherhood are the inheritance of a common humanity, and not of one country or one time, for neither political boundaries nor the unseen walls of different speech can circumscribe his fame, which, like the winds and the waves, gathers renewed vigor as it passes onward within the influence of alien zones until it finally reaches the "four corners of the world" and then—

> "The echoes roll from soul to soul,
> And grow forever and forever."

1817 to 1883:—A long, active, and highly honorable pilgrimage for Count Giovanni Battiste Ercolani. But now the great and solemn time was drawing nigh, for at the dawning of the year a treacherous malady (Epithelioma) had seized upon his system, and "the gentle science to whom nearly three-score years had been devoted, was now powerless to relieve her loving disciple." The last work of his life was completed during the few months that preceded the end. This is now before you in the form of his letters to Prof. Kölliker.

As Ercolani's first undertaking had been for the benefit of the world, so even was his final act, for his splendid library of manuscripts, codices, and rare editions—a fortune in itself—was modestly bestowed upon the city of his birth.

During his final illness he had on several occasions expressed a desire to be buried without unnecessary ceremony and expense, and, as the subject was taken up by the daily journals, he requested his nephew to communicate his wish to the Mayor, but the latter, mindful of Ercolani's worth replied:—"Bologna must do her duty, and I cannot prevent the Bolognese from showing the honor due to her illustrious citizen."

"It was heart-breaking," says Prof. Cocconi, "to witness the terrible suffering that preceded poor Ercolani's mortal overthrow, and which he endured with such heroic resignation."

Thus it fell that on the 16th of November, 1883, at the ripe age of sixty-four years, the gifted son of Leone and Lisi Ercolani paid the great debt of nature.

So ended "*The Labors of Hercules.*"

## Figures and Tables

**Figure f1:**